# An Open Label Trial to Assess Safety of Losartan for Treating Worsening Respiratory Illness in COVID-19

**DOI:** 10.3389/fmed.2021.630209

**Published:** 2021-02-17

**Authors:** Charles D. Bengtson, Robert N. Montgomery, Usman Nazir, Lewis Satterwhite, Michael D. Kim, Nathan C. Bahr, Mario Castro, Nathalie Baumlin, Matthias Salathe

**Affiliations:** ^1^Department of Internal Medicine, Division of Pulmonary, Critical Care and Sleep Medicine, University of Kansas Medical Center, Kansas City, KS, United States; ^2^Department of Biostatistics and Data Science, University of Kansas Medical Center, Kansas City, KS, United States; ^3^Department of Internal Medicine, Division of Infectious Diseases, University of Kansas Medical Center, Kansas City, KS, United States

**Keywords:** COVID−19, respiratory failure, inflammation, losartan, Bayesian, angiotensin receptor inhibitors

## Abstract

**Rationale:** Coronavirus disease 2019 (COVID-19) can cause disruption of the renin-angiotensin system in the lungs, possibly contributing to pulmonary capillary leakage. Thus, angiotensin receptor blockers (ARBs) may improve respiratory failure.

**Objective:** Assess safety of losartan for use in respiratory failure related to COVID-19 (NCT04335123).

**Methods:** Single arm, open label trial of losartan in those hospitalized with respiratory failure related to COVID-19. Oral losartan (25 mg daily for 3 days, then 50 mg) was administered from enrollment until day 14 or hospital discharge. A *post-hoc* external control group with patients who met all inclusion criteria was matched 1:1 to the treatment group using propensity scores for comparison.

**Measures:** Primary outcome was cumulative incidence of any adverse events. Secondary, explorative endpoints included measures of respiratory failure, length of stay and vital status.

**Results:** Of the 34 participants enrolled in the trial, 30 completed the study with a mean age SD of 53.8 ± 17.7 years and 17 males (57%). On losartan, 24/30 (80%) experienced an adverse event as opposed to 29/30 (97%) of controls, with a lower average number of adverse events on losartan relative to control (2.2 vs. 3.3). Using Poisson regression and controlling for age, sex, race, date of enrollment, disease severity at enrollment, and history of high-risk comorbidities, the incidence rate ratio of adverse events on losartan relative to control was 0.69 (95% CI: 0.49–0.97)

**Conclusions:** Losartan appeared safe for COVID-19-related acute respiratory compromise. To assess true efficacy, randomized trials are needed.

## Introduction

Since emergence of the severe acute respiratory syndrome coronavirus 2 (SARS-CoV-2), responsible for COVID-19, the global clinical and research community put forth great efforts to evaluate potential therapeutics. Particular attention has been given to repurposing previously developed or approved drugs with plausible mechanisms of benefit to expedite trials given the widespread and significant burden of this disease (1–3). A key question in treating COVID-19 is how to mitigate the development of respiratory failure–one potential answer is the use of angiotensin receptor blockers (ARBs).

Arguments against or for use of ARBs during COVID-19 were published in several opinion pieces (4–6). On the other hand, the premise for ARB use is based on scientific data assembled in multiple models of viral pneumonia. Like some other coronaviruses including SARS-CoV and HCoV-NL63 (7), SARS-CoV-2 infects cells by binding to angiotensin-converting enzyme 2, or ACE2 (8, 9), a protein expressed in the lung (10). The enzyme is thereby downregulated, causing dysregulation of the renin-angiotensin system that results in more angiotensin II (11) and less angiotensin-(1–7) (a vasodilator) (12, 13). This imbalance is expected to increase activation of the angiotensin II type I receptor (AT1R), located on type II alveolar cells, shown to mediate pulmonary capillary leak and alveolar damage (14, 15). In fact, elevated serum levels of angiotensin II in subjects with COVID-19 are correlated with higher viral load, disease severity, and respiratory failure (16, 17). However, these levels were outside the physiological range and determined using unvalidated assays, questioning these conclusions (18).

AT1R blockade or knockdown, while not consistent across all studies, was reported to be associated with decreased lung injury in some murine models of lung injury (12, 13). Furthermore, multiple publications showed no adverse effect of ARBs on plasma ACE2 activity (19–21). While some studies did not show any change in cellular expression of ACE2 with AT1R blockade (22, 23), others reported that AT1R blockade can increase ACE2 in renal and cardiac tissues of animal models (24–26). The latter could raise concerns about the possibility of increased viral entry and worse outcomes in COVID-19. However, perhaps paradoxically, ACE2 upregulation may be beneficial due to upregulation of angiotensin-(1-7) production as shown in at least one disease model (27). The possible benefit of ARBs for COVID-19 is also supported by multiple retrospective studies showing that patients on chronic ARB or ACE inhibitor therapy had similar or even less severity of disease (28–33). In addition, two recent randomized trials, BRACE-CORONA and REPLACE COVID, found that continuation of ACE inhibitors and ARBs was not associated with worse outcomes in hospitalized patients with COVID-19 (34–36). Furthermore, ARBs have anti-inflammatory properties, independent of their angiotensin receptor blocking ability (37–40).

Given the evidence supporting the potential benefit of ARBs in COVID-19, we conducted a single arm, open-label, externally controlled trial to determine the safety of using losartan *de novo* to treat respiratory failure caused by COVID-19. There is one preprint evaluating the use of ARBs to treat COVID-19 (NCT04355936), showing possibly positive results (41).

## Methods

### Trial Design and Oversight

We designed a single arm, open-label, dose-escalation trial of losartan in COVID-19. The trial was approved by the University of Kansas Medical Center Institutional Review Board and overseen by an independent data and safety monitoring board (DSMB). All participants underwent informed consent prior to study procedures. An interim safety analysis was done after five participants and 30 participants completed the study. As this study's primary outcome was safety no sample size calculations for efficacy were completed prior to enrollment. An investigational new drug exemption was obtained from the Food and Drug Administration for the use of losartan in this trial (NCT04335123). The full protocol can be found in the supplement.

### Participants

Consecutive admissions to the University of Kansas Hospital were screened for enrollment. Inclusion criteria included: >18 years of age, admission to the University of Kansas Hospital, a history of SARS-CoV-2 infection by polymerase chain reaction (PCR) and respiratory failure (supplemental oxygen use or a pulse oximetry reading of <94% on ambient air). Exclusion criteria included: pregnancy, use of ACE inhibitor or ARB prior to admission, chronic use of medications known to interact with losartan (non-steroidal anti-inflammatory drugs, potassium supplementation and aliskiren), prior intolerance to ARBs, respiratory failure due to a process other than COVID-19, kidney failure (creatinine clearance <30 ml/min or urine output <20 ml/h), serum potassium level >5.5 mmol/L, known renal artery stenosis, hypotension (systolic blood pressure >90 mmHg and diastolic blood pressure >60 mmHg; or for those on mechanical ventilation, a dose of norepinephrine >0.1 μg/kg/min (given the hemodynamic effects associated with sedation), history of left ventricular ejection fraction <35%, hepatic dysfunction (liver function tests >5 times upper limit of normal), meeting all inclusion criteria for >48 h prior to enrollment or other neurologic, psychiatric, neoplastic or endocrinologic disease judged by the investigator to interfere with participation in the trial.

### Study Procedures

Following informed consent, participants received 25 mg of losartan once daily for 3 days which, if not halted due to predefined criteria (see exclusions), was increased to 50 mg once daily. Losartan was continued for up to 14 days, until hospital discharge or if pre-defined parameters for holding losartan were met, whichever occurred first. Pre-defined parameters for holding losartan included the exclusion criteria listed above plus onset of skin rash without clear explanation or any change in monitoring parameter deemed significant and potentially related to losartan. If holding criteria parameter(s) improved during the study period, and were not felt to be related to medication, losartan was restarted at 25 mg once daily with dose escalation to 50 mg once daily. Study team members assessed daily clinical endpoints and protocol-defined adverse events (Supplementary Table 1). Laboratory monitoring was completed daily as per routine clinical care.

### Cytokine Analysis

Plasma samples were obtained at enrollment and study exit for cytokine analysis. They were aliquoted and stored at −80°C. Plasma levels of interleukin-6 (IL-6), interleukin-8 (IL-8) and tissue necrosis factor alpha (TNF-α) were measured by automated ELISA (ELLA microfluidic analyzer, ProteinSimple, San Jose, California USA). Samples were analyzed in triplicate with mean values used for analysis.

### Outcome Measures

The primary endpoint was the cumulative number of adverse events. Secondary, explorative endpoints included incidence of individual adverse events, days requiring supplemental oxygen during the study period, incidence of mechanical ventilation, days on mechanical ventilation during study period, intensive care unit (ICU) length of stay, hospital length of stay and vital status at hospital discharge. Additionally, the change in levels of cytokines were measured as an exploratory endpoint.

### *Post-hoc*, External Controls

A group of patients hospitalized at the University of Kansas Hospital with a confirmed diagnosis of COVID-19 were used as an external control group. This included a historical group of patients, who were admitted prior to the beginning of trial enrollment, and a parallel group of patients, who were admitted during the trial enrollment period but declined participation. All patients in the external control group met only all inclusion criteria. These patients were identified retrospectively, and their clinical data obtained through chart review. Data were collected from the date of admission through day 14 or hospital discharge, whichever occurred first. Adverse events and determination of clinical endpoints were assessed in a manner identical to those enrolled in the trial.

### Statistical Analysis

Before analysis, we used 1:1 nearest neighbor matching based on propensity scores to account for imbalance between the external control and losartan groups. The propensity score model was chosen based on graphical assessments of balance and overlap from a range of potential models and matching methods. This resulted in a final sample size for analysis of 60, with 30 in both the losartan and control group. The primary outcome was analyzed using a Poisson regression model with an offset for time in the study. The model was adjusted for age, sex, race, and history of high-risk comorbidities (obesity, hypertension and diabetes) as well as severity of disease which was determined by the type of oxygen support the subject received on the date of admission (room air, nasal cannula, non-invasive ventilation, invasive ventilation).

Model fit was assessed through diagnostic plots and 95% confidence intervals were calculated for all parameter estimates. Due to the relatively small sample and retrospective control group and as a sensitivity analysis, we re-analyzed the primary outcome using Bayesian Poisson regression models with skeptical priors. For the primary outcome we used a model with normal priors for the effect of losartan, N(0,0.50), and for all other covariates N(0,1). The prior for losartan is centered at 0, no effect, and on the incidence rate ratio scale put 95% of the prior probability on values between 0.38 and 2.67: this will essentially shrink large effects closer to 0. We also re-fit both the Poisson and Bayesian Poisson models with an effect for having received other effective COVID-19 therapies (remdesivir and dexamethasone) at any point during their admission.

Days requiring supplemental oxygen, days requiring mechanical ventilation, length of stay in the ICU and hospital length of stay were analyzed using Poisson regression models with the same covariates and priors as the primary endpoint (no offset was used for overall length of stay). Odds of progression to mechanical ventilation (binary) and vital status (alive or dead, binary) at discharge were analyzed using logistic regression models with the same covariates as the primary endpoint. For incidence of mechanical ventilation, those who entered the study on mechanical ventilation were excluded from analysis and we also included an indicator variable (before/after date of first enrollment in losartan group) given potential changes in the threshold for escalation to mechanical ventilation over time. For the exploratory endpoint of change in plasma cytokine levels, a Wilcoxon signed-rank test was used. All analyses were conducted using R (R Core Team), Stan (42), rstanarm (43), or Prism (GraphPad Software, San Diego CA, USA).

## Results

### Participants and Non-randomized Controls

**Figure 1:**
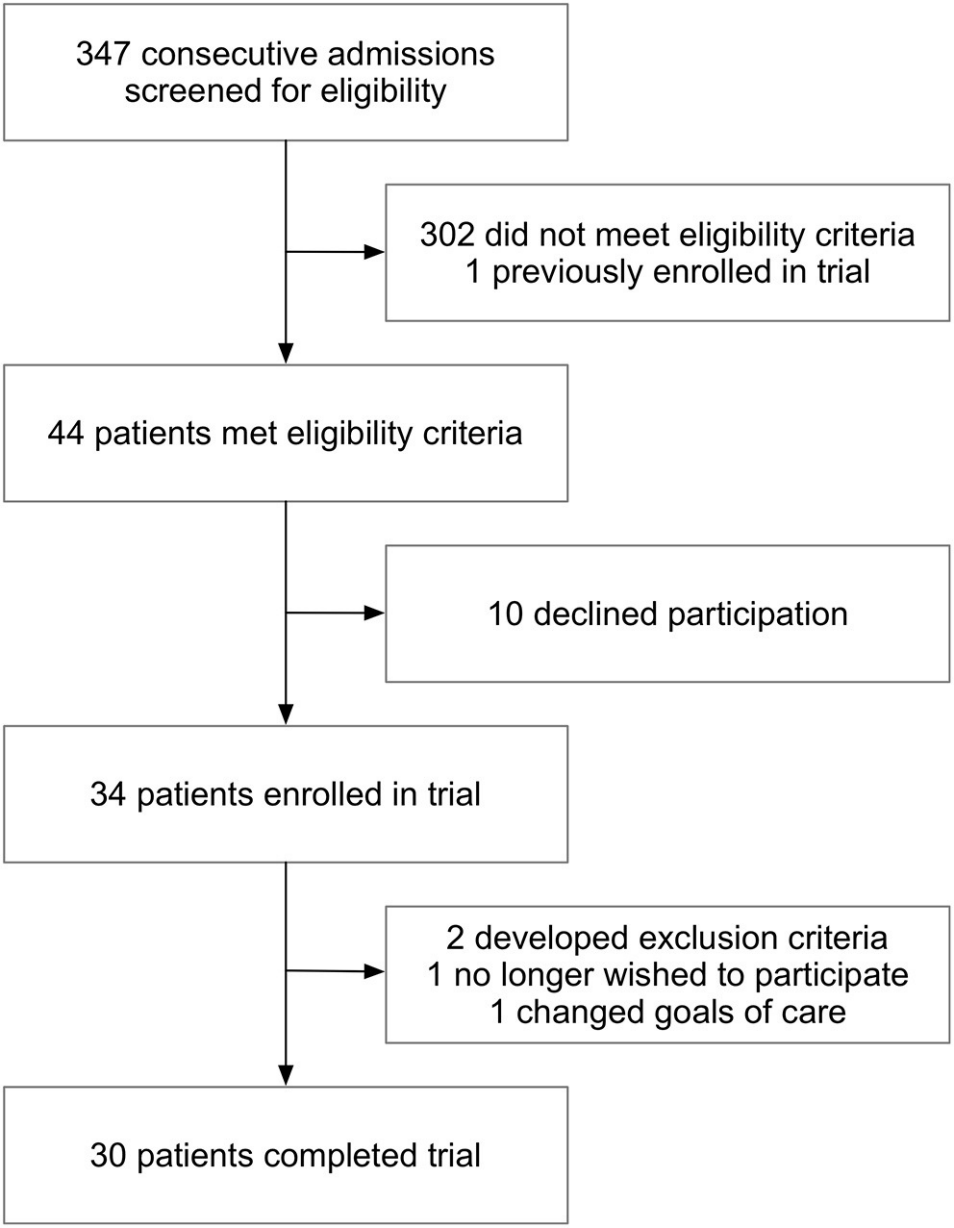
Screening and enrollment. Out of 347 admissions screened, 44 met criteria for enrollment into the losartan group. Of the 34 participants that were enrolled into the trial, there were 30 who completed the study. Two participants were withdrawn for development of exclusion criteria after informed consent (hypotension and prior use of ACE inhibitor or ARB that was not known on enrollment), one chose to withdraw and one changed goals of care. A total of 30 participants completed all study procedures.

Beginning April 2nd, 2020, consecutive admissions with suspected or confirmed COVID-19 were screened for enrollment (Figure 1). Of 44 patients who met eligibility criteria, 34 were prospectively enrolled in the trial. There were 30 participants who completed the study. Reasons for not completing the study included: development of exclusion criteria after enrollment (2), withdrawal due to participant choice (1) and change in goals of care to comfort only (1). For external controls, 118 other COVID-19 patients were identified who were hospitalized prior to and during the trial, had a positive SARS-CoV-2 PCR test and were not enrolled in the losartan group. Of these patients, 46 met all inclusion criteria, and using propensity score matching we kept 30 of these 46 patients as the final control group. For both participants and non-randomized controls, data from subsequent admissions was not collected.

There were differences in baseline characteristics between groups (Table 1). The losartan group had greater proportions of chronic respiratory disease (47 vs. 7%), and a larger number of White patients were in the losartan group (50 vs. 20%). Adjunct therapies were quantified between groups. A higher proportion of those in the losartan group received remdesivir (7/30 vs. 0/30). The frequency of other COVID-19 therapies including hydroxychloroquine, tocilizumab and lopinavir/ritonavir was approximately balanced between groups (Supplementary Table 2).

**Table 1:** Baseline demographics and clinical characteristics.

	**Losartan (*n* = 30)**	**Control (*n* = 30)**	***P*-value**
Age (years)^a^	54.2 (15.1)	53.8 (15.5)	0.92
Sex (male sex %)	57	53	0.99
Race %			<0.01
White	50	27	
Black	27	30	
Other	23	43	
Hypertension %	50	47	0.99
Diabetes %	30	40	0.59
History of malignancy %	10	3	0.61
Chronic respiratory disease %^b^	47	7	<0.01
Chronic kidney disease %	13	7	0.67
BMI Mean (SD)	33.5 (7.6)	32.3 (9.6)	0.60
Severity status %			0.26
Room air	20	36	
Nasal cannula	50	47	
Non-invasive ventilation^c^	17	3	
Invasive ventilation	13	13	

a *Mean (standard deviation)*.

b *Defined as a diagnosis of chronic obstructive pulmonary disease, asthma or interstitial lung disease*.

c *Includes both heated high flow nasal cannula and non-invasive positive pressure ventilation*.

Interim review by the DSMB found no safety concerns after five and allowed for completion of the study after the second review; 30 participants completed study procedures.

### Primary Outcome

**Figure 2:**
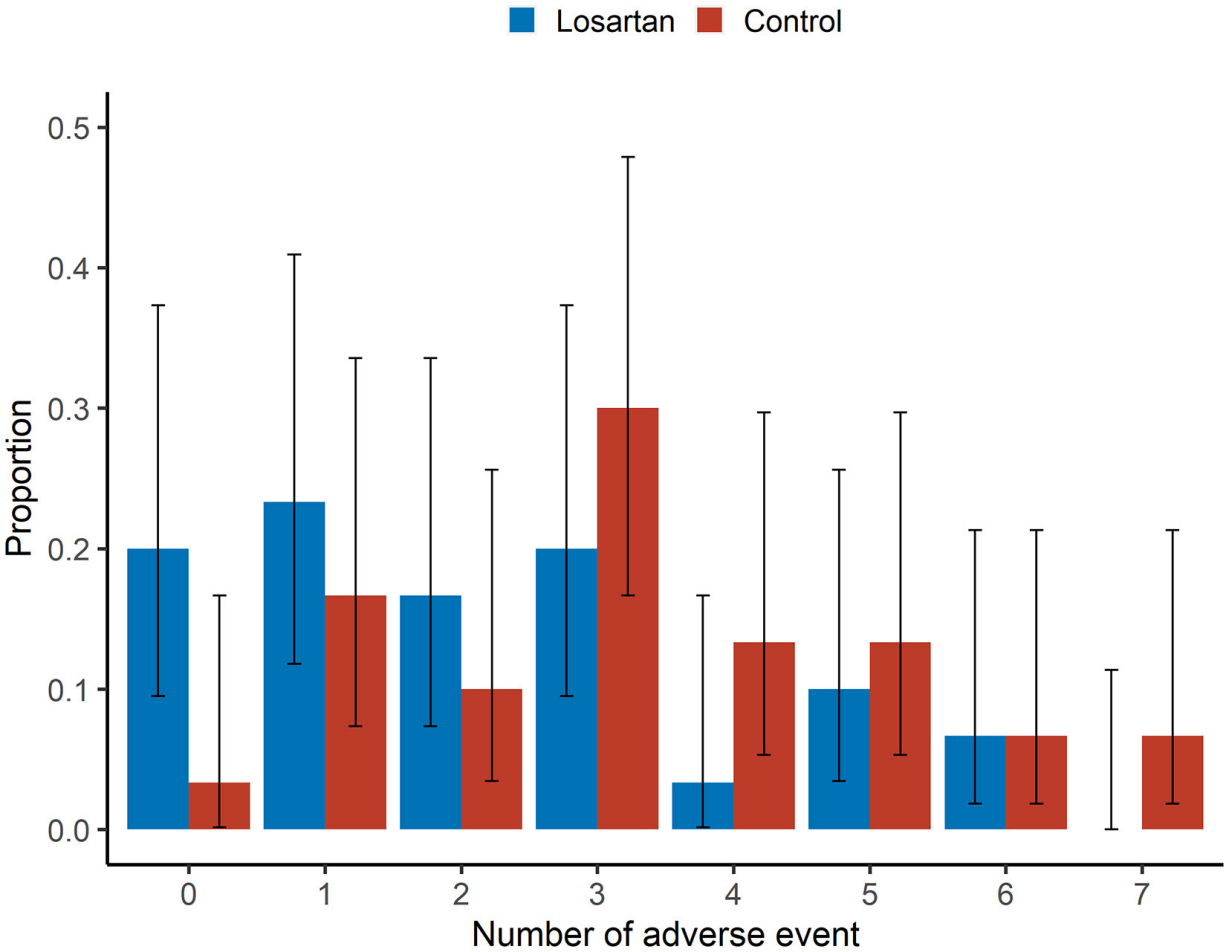
Adverse events. Sample proportion means and 95% confidence intervals of adverse event rates between losartan and control groups. There was a greater proportion of those in the losartan group experiencing no adverse events (20 vs. 3.3%).This analysis was adjusted for age, sex, race, disease severity at enrollment (ambient air, nasal cannula, non-invasive ventilation or invasive ventilation), presence of high-risk comorbidities with an offset for number of days in the study. There was no adjustment for duration or severity of adverse events.

A greater proportion of those in the control group experienced at least one adverse event (29/30, 97% vs. 24/30, 80%; Figure 2, Table 2). Controlling for age, sex, race, date of and severity of disease at enrollment, and history of high-risk comorbidities, we estimated the incidence rate ratio of adverse events relative to the comparator group (IRR—losartan relative control) to be 0.69, 95% confidence interval (0.49, 0.97) (Table 2). In the Bayesian Poisson sensitivity analysis, we estimated the IRR to be 0.72, with 95% credible interval (0.50, 0.96; Supplementary Table 3). Additionally, the Bayesian model allowed us to calculate the posterior probability that the losartan group had a lower adverse event rate (i.e., IRR < 0), something the frequentist approach does not allow: the posterior probability that losartan was effective at reducing adverse events was 98%.

**Table 2A:** Primary outcome model.

	**Estimated coefficient**	**Standard deviation**	**IRR^**a**^ (95% CI)**
**Outcome of interest**			
Treatment (Losartan)	−0.37	0.17	0.69 (0.49, 0.97)
**Covariates**			
Age (years)	−0.004	0.006	1.0 (0.99, 1.01)
Sex (Male)	0.10	0.17	1.11 (0.79, 1.56)
Race (White)	−0.22	0.19	0.81 (0.55, 1.16)
Severity^b^	0.005	0.08	1.01 (0.85, 1.18)
High risk comorbidities^c^	−0.02	0.09	0.98 (0.82, 1.18)

a *IRR, incidence rate ratio*.

b *Respiratory status at enrollment; includes ambient air, nasal cannula, non-invasive ventilation and invasive ventilation*.

c *High risk comorbidities include one or more of the following diagnoses: Diabetes, BMI >30 and/or hypertension*.

**Table 2B:** Proportion of subjects experiencing number of adverse events.

	**Number of adverse events**							
**Group**	**0**	**1**	**2**	**3**	**4**	**5**	**6**	**7**
Losartan (%)	20	23	17	20	3	10	7	0
Control (%)	3	17	10	30	13	13	7	7

### Secondary Outcomes

**Figure 3:**
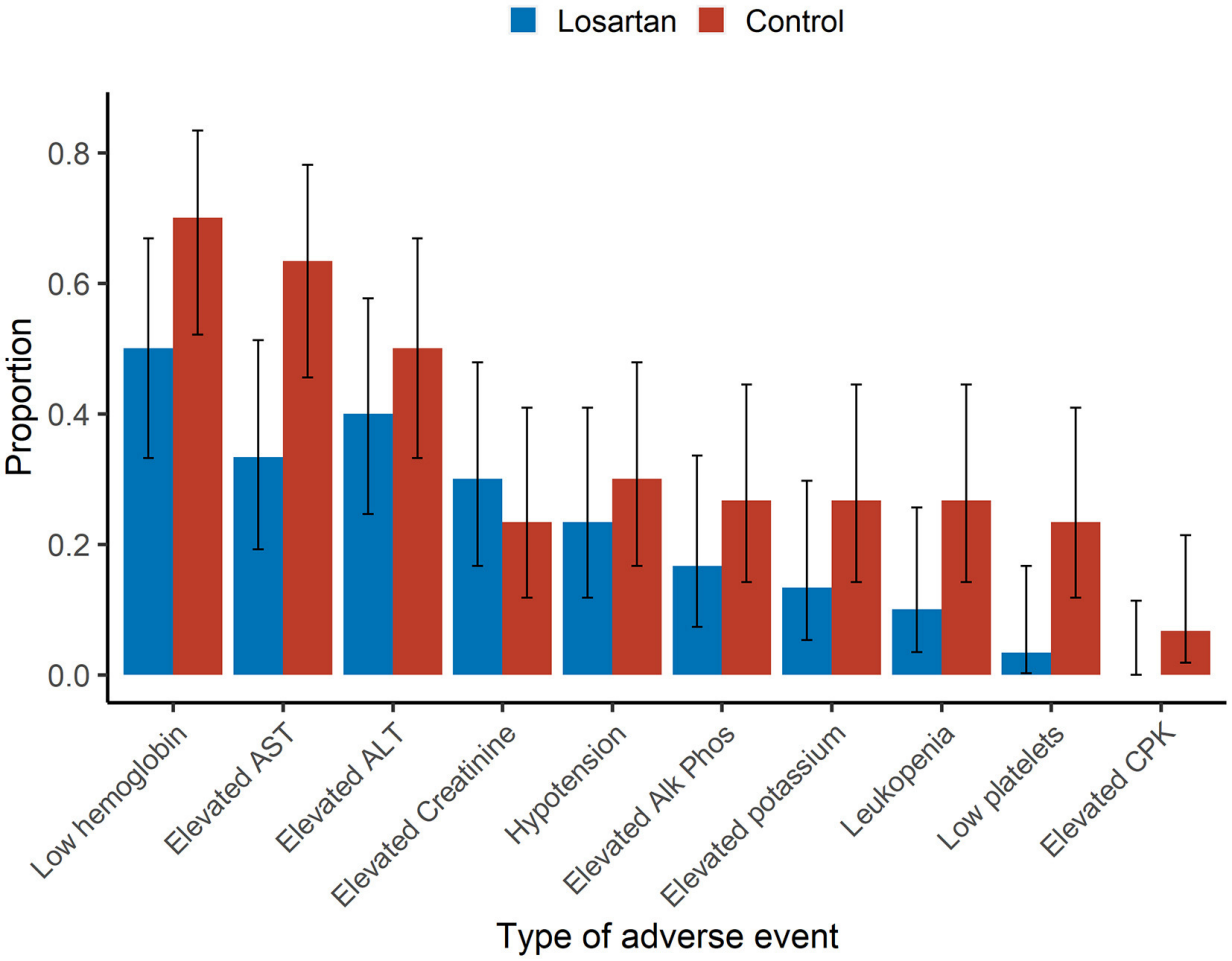
Type of adverse events. Sample proportions means and 95% confidence intervals of individual adverse event proportions among participants. Adverse events were assessed daily and classified per protocol. Aside from elevated creatinine (30 vs. 23%), the estimated event proportions for losartan were lower for each individual adverse event. There was a significantly lower estimated proportion of the adverse event elevated aspartate aminotransferase (AST) 33 vs. 63% (*p* = 0.04) in the losartan group compared to control.

The mean number of adverse events per patient was 2.2 on losartan vs. 3.3 in controls. Additionally, there were more participants in the losartan group who experienced 0 adverse events compared to controls (6/30 vs. 1/30) and fewer participants in losartan relative to control who experienced seven adverse events, the highest observed, 0/30 vs. 2/30 (Figure 2). There was also evidence of different proportions of specific adverse events (Figure 3), with those in the losartan group experiencing fewer adverse events on all specific types except for elevated creatinine (30% losartan vs. 23% control). The largest difference was with the proportion of elevated aspartate aminotransferase (33% losartan vs. 63% control). However, only the difference in the proportion of AST adverse events reached statistical significance and is of unclear clinical significance.

After controlling for age, sex, race, severity of disease, history of high-risk comorbidities and date of enrollment, we did not observe a strong effect on incidence of mechanical ventilation, length of stay in the ICU, overall hospital length of stay, days requiring supplemental oxygen, days requiring mechanical ventilation or mortality status at the end of study. However, the effect of losartan was estimated to be beneficial on all these endpoints. For instance, the incidence of death (1/30 vs. 3/30) and of mechanical ventilation (2/17 vs. 5/17) was lower in losartan relative to control; however, the event rates were too small and thus our estimates were too imprecise to draw conclusions.

There were five participants who met criteria for holding losartan due to: elevated creatinine (3), elevated aminotransferases (1) and hypotension (1). Of those five participants, four were able to tolerate resumption of losartan and reached the target dose of 50 mg.

### Plasma Cytokine Levels

There were 27 participants in the losartan group with study plasma samples at enrollment and end of study available for analysis. A significant decrease in plasma levels of IL-6 (*p* = 0.003) occurred during the study period while levels of IL-8 and TNF-α remained relatively unchanged. No plasma was collected from the control group (Supplemental Figure 1).

## Discussion

In this open label, non-randomized trial which utilized an external, *post-hoc* control group, we evaluated the safety of using losartan to treat respiratory failure related to COVID-19. We found that the rate of adverse events was significantly less in those treated with losartan compared to an external control group.

The results of our study are in line with multiple observational studies demonstrating that prior use of ACE inhibitors and ARBs were not associated with worse outcomes in COVID-19, which was a concern early during the pandemic (32, 44). In addition, they are consistent with a preprint indicating that telmisartan might be beneficial for patients, even though these outcomes were length of stay and related to inflammatory parameters with no difference in escalation to mechanical ventilation (41). The results also align with those of the BRACE-CORONA REPLACE COVID trials, which found that continuation of ACE inhibitors and ARBs, vs. cessation, was not associated with worse outcomes in those hospitalized with COVID-19 (34–36).

### Limitations

There are several limitations to consider when interpreting the results of our study, foremost the use of external controls and lack of randomization. Utilizing an open label design with external controls allowed us to address, in a timely manner, a clinically relevant concern regarding the safety of ARBs in COVID-19. The external controls included a historical and parallel group, and while propensity score-based matching resulted in better balance and overlap, we still had imbalance on several variables and did not achieve perfect overlap. Additionally, as external controls may have met some exclusion criteria, some degree of selection bias still remains. To mitigate this fact, we utilized multivariable models to adjust for baseline characteristics and factors that are known to be associated with worse outcomes in COVID-19 (45–47). We also performed many sensitivity analyses and observed small differences between these models and the original analysis. However, despite these attempts, there is potential for confounding and/ or bias in the estimated effects. It is also unclear what effect collider bias had on the incidence of adverse events since many of them are known to be caused by COVID-19 (48). Additionally, there may be a time-dependent bias given that enrollment occurred at different time points in participants disease process which was not accounted for in the final analysis.

After our trial began, remdesivir and dexamethasone demonstrated efficacy in improving outcomes in COVID-19 (49, 50). There was a higher frequency of use of remdesivir in the losartan group with approximate balance for dexamethasone (Supplementary Table 2). When adjusting for this, the variation in our estimates increased, but the actual point estimates were largely unchanged as seen in Supplementary Table 3 (50). This is possibly consistent with newer data finding little effects of remdesivir in more advanced disease (51).

We chose to use 50 mg as the target dose of losartan. While this dose is commonly used for hypertension, it is possible that higher doses could be more efficacious when treating respiratory failure in COVID-19. Pharmacokinetic studies in healthy volunteers at higher doses have demonstrated that twice daily dosing provides more effective blockade of AT1R to help restore the equilibrium between the Ang II and angiotensin-(1-7) pathways thus attenuating lung injury caused by SARS-CoV-2 (52, 53). Given its alternative mechanisms of action, demonstrated safety and ease of access, losartan remains a potential adjunct therapy for the treatment of respiratory failure related to COVID-19. In fact, there are ongoing randomized controlled trials evaluating losartan in patients with COVID-19 that are designed and powered to detect evidence of efficacy if present (NCT04312003, NCT04311177).

## Data Availability Statement

The raw data supporting the conclusions of this article will be made available by the authors, without undue reservation.

## Ethics Statement

The studies involving human participants were reviewed and approved by University of Kansas Medical Center. The patients/participants/proxies provided their written informed consent to participate in this study.

## Author Contributions

MS, CB, UN, LS, NB, MK, and MC: concept and protocol development. MS, CB, and NB: participant consent and study procedures. CB: electronic database design and maintenance. MS, CB, and RM: statistical plan and analysis. MS, CB, and RM: manuscript first draft. MS, CB, RM, UN, LS, MK, NB, MC, and NB: manuscript review and revision. All authors contributed to the article and approved the submitted version.

## Conflict of Interest

CB reported receiving grant support from the NIH National Center for Advancing Translational Sciences (NCATS) and Cystic Fibrosis Foundation (CFF) during the conduct of the study. NB reported receiving grant support from the NIH National Institute of Neurologic Disease and Stroke during the conduct of the study. MC reported receiving University grant support from the NIH, American Lung Association and PCORI; pharmaceutical grant support from AstraZeneca, GSK, Novartis, Pulmatrix, Sanofi-Aventis and Shinogi; is a consult for Genentech, Teva, Sanofi-Aventis and Novartis; is a speaker for AstraZeneca, Genentech, GSK, Regeneron, Sanofi and Teva; receives royalties from Elsevier – all unrelated to the conduct of this study. MS reported receiving grant support from the NIH NHLBI, James and Ester King Biomedical Research Program, FAMRI and CFF during the conduct of the study. The remaining authors declare that the research was conducted in the absence of any commercial or financial relationships that could be construed as a potential conflict of interest.
