# Supplementary material for: An Open Label Trial to Assess Safety of Losartan for Treating Worsening Respiratory Illness in COVID-19
Source: Front Med (Lausanne). 2021 Feb 17;8:630209. doi: 10.3389/fmed.2021.630209 (PMC7926174; doi:10.3389/fmed.2021.630209)
Supplement: Supplementary file 1 [file Data_Sheet_1.PDF]

Supplemental Materials to

An open label trial to assess safety of losartan for treating worsening respiratory illness in COVID-19

Charles D. Bengtson, M.D., Robert N. Montgomery, Ph.D., Usman Nazir, M.D., Lewis Satterwhite, M.D., Michael D. Kim, Ph.D., Nathan C. Bahr, M.D., Mario Castro, M.D., M.P.H., Nathalie Baumlin, M.B.A., Matthias Salathe, M.D.

Author Affiliations:

From the University of Kansas Medical Center, Department of Internal Medicine, Division of Pulmonary, Critical Care and Sleep Medicine (C.D.B., U.N., L.S., M.D.K., M.S.) and Division of Infectious Diseases (N.B.); and the University of Kansas Medical Center, Department of Biostatistics and Data Science (R.N.M.).

Correspondence:

Matthias Salathe, Department of Internal Medicine, University of Kansas Medical Center, 3901 Rainbow Blvd, 4032 Delp, MS 1022, Kansas City, KS 66160, USA. Email: msalathe@kumc.edu

Collaborators:

Investigational Pharmacy Support: Barbara Couldry, Kristen Filby and Rocio Lager
Data and Safety Monitoring Committee: Nathan Bahr (chairperson), Andreas Schmid, Wissam El Atrouni, Jason Glenn, John Chen, Carolina Aguilar and Miranda Handke)
Medical Monitor: Mario Castro

Authorship Contributions:
Concept and protocol development: MS, CDB, UN, LS, NB, MDK and MC
IND exemption holder: MS
Participant consent and study procedures: MS, CDB and NB
Electronic database (REDCap) design and maintenance: CDB
Statistical plan and analysis: MS, CDB and RNM
Manuscript first draft: MS, CDB and RNM
Manuscript review and revision: MS, CDB, RNM, UN, LS, MDK, NCB, MC and NB

MS is the overall guarantor of the manuscript; all authors reviewed and gave final approval.

Plasma sample preparation for Cytokine analysis using Ella system from ProteinSimple
Blood samples were collected in EDTA tubes and processed within 6h after collection. The EDTA tubes were spun for 15 minutes at 2000 x g and 4 °C and the supernatants (plasma) were aliquoted and stored at -80 °C. Frozen samples were thawed and 30 µL was diluted with 30 µL of sample diluent. Fifty microliters of the diluted samples were pipetted into the cartridge. The cartridges were loaded and run into Ella following manufacturer’s instructions.

Table S1: Adverse event criteria and grading

Adverse event	Grade 1	Grade 2	Grade 3	Grade 4	Grade 5
Hemoglobin	Hemoglobin (Hgb) <LLN - 10.0 g/dL; <LLN - 6.2 mmol/L; <LLN - 100 g/L Follow up	Hgb <10.0 - 8.0 g/dL; <6.2 - 4.9 mmol/L; <100 - 80g/L ; follow up	Hgb <8.0 - 6.5 g/dL; <4.9 - 4.0 mmol/L; <80 - 65 g/L; transfusion indicated; stop losartan, follow up	Life-threatening consequences; urgent intervention indicated; stop losartan	Death
Leukocytes	<LLN - 3000/mm3; <LLN - 3.0 x 10e9 /L; follow closely	<3000 - 2000/mm3; <3.0 - 2.0 x 10e9 /L; stop losartan; follow up	<2000 - 1000/mm3; <2.0 - 1.0 x 10e9 /L; stop losartan; follow up and further investigation	<1000/mm3; <1.0 x 10e9 /L; stop losartan; follow up and further investigation	
Platelets	<LLN - 75,000/mm3; <LLN - 75.0 x 10e9 /L; follow closely	<75,000 - 50,000/mm3; <75.0 - 50.0 x 10e9 /L; follow closely	<50,000 - 25,000/mm3; <50.0 - 25.0 x 10e9 /L; follow closely, stop losartan,	<25,000/mm3; <25.0 x 10e9 /L; follow closely, stop losartan, transfer to ICU	
Angioedema	N/A	N/A	Angioedema occurs – mild: stop losartan, transfer to ICU	Angioedema occurs – severe tongue swelling: stop losartan, transfer to ICU	Death
Hypotension (for participants in ICU on sedation for mechanical ventilation ONLY)	Initiation of vasopressors	Increase in vasopressor dose to >0.1; stop losartan, follow up	Persistent hypotension without alternative cause; stop losartan, follow up	Irreversible end organ damage related to hypotension; stop losartan, follow up	Death
Hypotension (for participants NOT in ICU on sedation for mechanical ventilation)	Asymptomatic, intervention not indicated	Non-urgent medical intervention indicated; stop losartan, follow up	Urgent medical intervention or transfer to ICU indicated; stop losartan, follow up	Life-threatening and urgent intervention indicated; losartan, follow up	Death
Creatinine	>1 - 1.5 x baseline; >ULN - 1.5 x ULN; follow up	>1.5 - 3.0 x baseline; >1.5 - 3.0 x ULN; close follow up	>3.0 baseline; >3.0 - 6.0 x ULN; stop losartan, nephrology intervention	>6.0 x ULN; stop losartan, nephrology intervention	
Potassium	>ULN to 5.5 mmol/L; monitor	>5.5 - 6.0 mmol/L, stop losartan, close follow up	>6.0 - 7.0 mmol/L; urgent intervention indicated, stop losartan, intervention	>7.0 mmol/L; life-threatening consequences; stop losartan, intervention	

AST, ALT, alkaline phosphatase	>ULN - 3.0 x ULN; monitor closely	Asymptomatic with AST,ALT, alk.P. >3.0 -5.0 x ULN; >3 x ULN with the appearance of worsening of fatigue, nausea, vomiting, right upper quadrant pain or tenderness, fever, rash, or eosinophilia or bilirubin elevation, monitor	>5.0 - 20.0 x ULN; >5 x ULN for >2 weeks; stop losartan, monitor	>20.0 x ULN; stop losartan, monitor, other interventions	
CPK[#]	>ULN - 2.5 x ULN; monitor closely	>2.5 x ULN - 5 x ULN; stop losartan, monitor closely	>5 x ULN - 10 x ULN; stop losartan, monitor closely	>10 x ULN; stop losartan, monitor closely, possible admission with other abnormalities	

[#] Only done when clinically indicated

Table S2 - Frequency of adjunct therapies for COVID-19[a]

	Group		
Antiviral medications	Losartan (%)	Control (%)	P-value
Hydroxychloroquine	18 (60)	21 (70)	0.59
Dexamethasone	4 (13)	2 (7)	0.67
Remdesivir	7 (23)	0 (0)	0.02
Tocilizumab	3 (10)	5 (17)	0.71
Lopinavir/ritonavir	1(3)	1 (3)	1.0

a. Any use during hospital admission

Table S3 Estimated effects of Losartan for primary outcome in sensitivity analyses

Analysis	Estimated Coefficient	Standard Deviation	Incidence Rate Ratio	95% CI for IRR
Original analysis	-0.37	0.17	0.69	(0.49, 0.97)
Bayesian Poisson regression[a]	-0.33	0.16	0.72	(0.50, 0.96)
Adjunct therapies[b] (remdesivir/dexamethasone)	-0.31	0.18	0.73	(0.52, 1.03)
Bayesian adjunct therapies[c] (remdesivir/dexamethasone)	-0.32	0.17	0.73	(0.50, 1.0)

a. Bayesian logistic regression was done using the same data set matched by propensity scores. Normal priors were put on all covariates, N(0,1) for all explanatory variables except for losartan, which had a N(0, 0.50) prior. This is a skeptical prior, in that it gives more prior probability to smaller effects (between about 0.38, and 2.67) on the odds ratio scale. This shrinks effects back to 0 (1 on the OR scale). The CI for this row is a 95% highest density posterior credible interval.

b. This model includes a covariate for whether participants were on either remdesivir or dexamethasone at any time during hospital stay

c. Bayesian model with an effect for dexamethasone and/or remdesivir

Figure S1

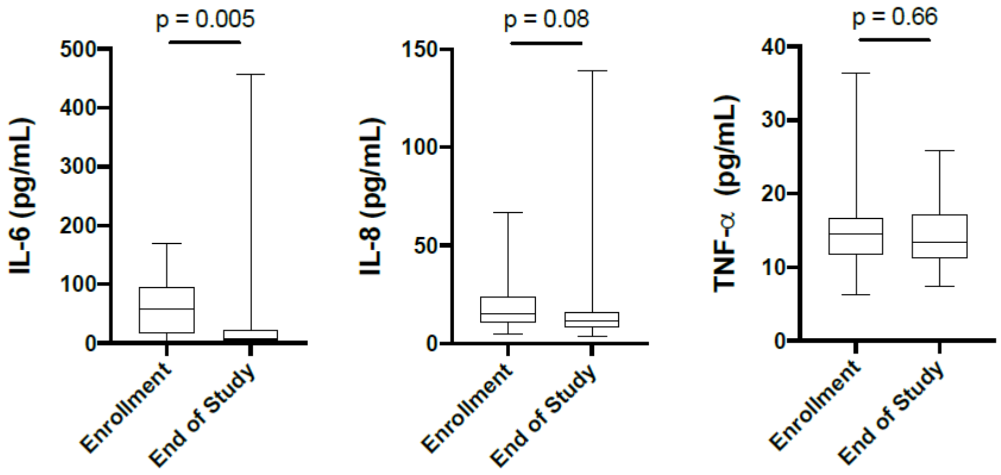

Figure S1: Change in plasma cytokine levels in those treated with losartan. Levels of plasma interleukin-6 (IL-6), interleukin-8 (IL-8) and tissue necrosis factor alpha (TNF-α) were measures via automated enzyme-linked immunosorbent assay. There was a significant decline in the level of IL-6 during the study period in those treated with losartan as assessed by the Wilcoxon rank-sum test (median 57.6 vs. 7.26 pg/mL, p = 0.005). There was no significant change in the levels of IL-8 (15.8 vs. 11.5 pg/mL, p = 0.08) or TNF-α (14.5 vs. 13.4 pg/mL, p = 0.66).
